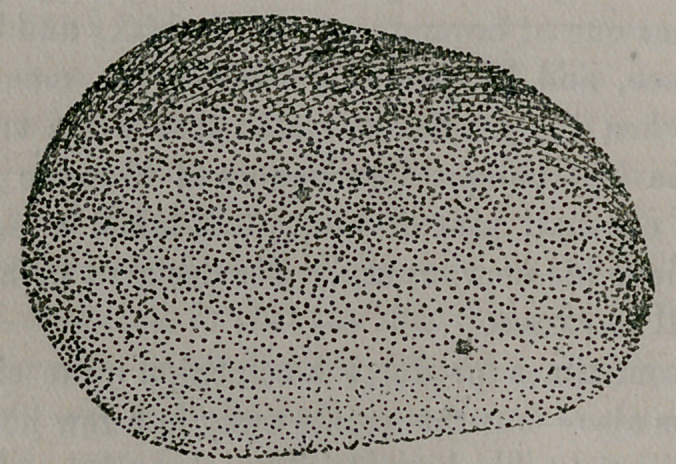# A Case of Calculus

**Published:** 1904-12

**Authors:** W. L. Champion

**Affiliations:** Atlanta, Ga.


### A CASE OF CALCULUS.

BY W. L. CHAMPION, M.D.,
ATLANTA, GA.

The accompanying picture is a likeness of a phosphatic stone removed from the bladder of J. R. A., on June 23d. The patient was a Caucasian, married, age twenty-five. He stated that for the past four or five years he had been troubled with frequent micturition, accompanied with pain. When he came to me he was very much emaciated, nervous, and in a pitiable condition on account of the suffering he was constantly undergoing. He had been examined and treated on several occasions for kidney trouble, cystitis and stricture. Any instrument inserted into the bladder would immediately come in contact with the stone, and the fact of a foreign body being present easily recognized. I present this case to show how careless some practitioners are in examining cases they treat. This man had passed through the hands of several with stone in his bladder weighing two ounces and as large as a hen egg.

I removed it through a suprapubic opening, and completely closed the bladder and abdominal openings and placed a soft rubber catheter in bladder through the urethra. The catheter was fastened with adhesive strips and allowed to remain several days.

The bladder was irrigated once daily with boric acid solution. Urotropin was used to correct the phosphatic condition of the urine. The bladder and abdominal incisions healed nicely; the patient was out of bed in sixteen days, and able to return home in three weeks.

---

### STERILE WATER ANESTHESIA IN OPERATIONS UPON THE RECTUM AND ANUS.

S. G. Gant (*Medical Record*, October 29, 1904) describes the excellent results he has had in substituting plain sterile water for cocaine and other solutions that are in vogue for the production of local anesthesia. The author has been able by its means to operate upon most rectal cases without a general anesthetic or sending them to the hospital. Anesthesia apparently is produced merely by the pressure of the fluid on the nerve terminals in the tissues, and sufficient water should be introduced thoroughly to distend the tissues, causing them to become anemic and assume a glassy, whitish appearance, when anesthesia immediately follows. This distention does not require a large amount of water, from ten minims to half a drachm only being necessary for small hemorrhoidal tumors, and from one-half to four drachms in more extensive operations. In introducing the water it is not necessary to use more force than is usually employed in making the ordinary hypodermic injection. In conclusion the writer states that, while anesthesia by the injection of sterile water is not effective and can not be applied in all major operations, he has employed it, to the exclusion of general and local medicinal anesthetics, in nearly all his operations upon the rectum (for hemorrhoids, fistula, fissures, etc.), and with such gratifying results that he heartily recommends its thorough trial by other surgeons for operations in the anorectal and other regions of the body.

## Figures and Tables

**Figure f1:**